# Deployment Risk and Resilience Model Applied to Military Children

**DOI:** 10.5964/ejop.1694

**Published:** 2022-05-31

**Authors:** Renato Pessoa dos Santos, Rita Francisco, Maria T. Ribeiro

**Affiliations:** 1 Academia Militar, Exército Português, Lisbon, Portugal; 2 CRC-W: Catolica Research Centre for Psychological, Family and Social Wellbeing, Universidade Católica Portuguesa, Lisbon, Portugal; 3 Faculdade de Ciências Humanas, Universidade Católica Portuguesa, Lisbon, Portugal; 4 Faculdade de Psicologia, Centro de Investigação em Ciências Psicológicas, Universidade de Lisboa, Lisbon, Portugal; California University of Pennsylvania, California, PA, USA

**Keywords:** military children, deployment cycle, coping strategies, risk and resilience model, resilient outcomes

## Abstract

This exploratory study investigates the impact of a military mission on Portuguese families, specifically on children. Although most research seeks the negative consequences of this lived experience, through the “Deployment Risk and Resilience Model” the present study intends to explore if this period can also be an opportunity for military’s children to grow and become more resilient. Aiming to express freely their lived and felt stories about the phenomenon under study, semi-structured interviews were conducted with 22 children of the service members of the Portuguese Army, aged between 8 and 21 years old. The results of the thematic analysis indicated that the most critical moments of the mission were the notification period, the last days before the departure of the service member, and the deployment. The preparation of activities for the service members’ absence in the pre-deployment and the increase of tasks to be carried out, during the deployment, were the most referenced changes. In the post-deployment, children perceived a rapid readjustment of the family system. Despite the military's children's difficulties in readjusting during the mission, they reported that the feelings of closeness to the nuclear family, increased responsibility, and personal growth were positive results experienced. It would be interesting to extend similar studies within family systems, as in other branches of the armed forces. As practical implications, the findings of our pioneering study may significantly contribute to the construction of programs and/or actions that promote a possible growth in the personal resilience of the children of Portuguese service members, and not only the recovery of the state prior to the mission.

Generally, an international military mission is organized in three phases: pre-deployment, which begins with the notification of the deployment until the departure of the service member to the Theater of Operations (TO); the deployment, period that corresponds to the time during which the service member, men or women, is in the TO, geographically separated from their family (usually 6 months in Portuguese missions); and post-deployment, which begins when the service member returns home (e.g., Paley, Lester, & Mogil, 2013). Each phase of the mission is associated with particular stress-inducing circumstances that influence the family system of the deployed military (Pincus, House, Christenson, & Alder, 2001), including fear and apprehension that the service member can be injured, or even die during the deployment phase. To this challenge other stress inducers are added, such as, increased workload (e.g., taking care of household chores, taking children to school and being in charge of bills), disturbing news and rumors and/or the possibility of difficulties in the communication with the service member, for example, lack of Internet or lack of delayed communication, such as letters or parcels (e.g., Andres & Coulthard, 2015; Bóia, Marques, Francisco, Ribeiro, & Santos, 2018).

Studies have shown that separations caused by the service members participating in a mission have an impact on their children, considering that emotional and behavioral adverse effects are frequently reported (e.g., Chandra et al., 2010). Andres and Coulthard (2015) report that the negative consequences inherent to a mission may be related to the absence of the military caregiver rather than the duration of the separation or the type of TO where the father was deployed to. On the other hand, Chandra et al. (2010) mention that there is a positive relationship between the difficulties in dealing with domestic and school responsibilities, and the duration of the deployment phase. Also, some studies suggest that the existence of the fear that something can happen to the military caregiver is amplified by the perception of the dangerousness of the TO (e.g., Kelley, 1994). Other studies mention that the children of the deployed military can experience an emotional cycle similar to that of their mothers, so showing externalization problems, such as aggressiveness and somatic complaints, and internalization problems, such as, crying, sadness, anger, and anxiety (e.g., Kelley, 1994; Knobloch, Pusateri, Ebata, & McGlaughlin, 2015). Thus, these factors can influence their daily lives (e.g., Flake, Davis, Johnson, & Middleton, 2009; Knobloch et al., 2015; Lester et al., 2010). Many studies focus on the negative effects of missions, leaving possible positive effects unanswered (Park, 2011). However, this (re)adjustment period can also be an opportunity for personal growth, promoting individual and family resilience (e.g., Knobloch et al., 2015; Park, 2011). In the study conducted by Knobloch et al. (2015), the children of deployed service members reported that during the mission they felt more responsibilities, family traditions were lost, there were changes in routines or they felt the family incomplete, among other negative consequences. However, constructive and beneficial results were also reported, such as the feeling of greater family cohesion, a perception of greater independence, the notion of enjoying new activities with the deployed service member, and having the feeling that they are better prepared for future missions (Knobloch et al., 2015).

## Children’s Coping and Resilience

Although a military mission presents risks, especially for some families (Park, 2011), for example, coping with disturbances to everyday activities, emotional problems, heightened family conflict (Knobloch et al., 2015), many children and military families show resilience and growth. Resilient children are those who, after exposure to risk factors, can overcome those risks, so avoiding negative outcome, such as behavioral problems, psychological imbalances and/or school difficulties (Rak & Patterson, 1996). Considered more dynamic than static, varying with situations and over time, resilience can be seen as a result, a response, or a process (Silgo & Mora, 2013). Richardson, Neiger, Jensen, and Kumpfer, (1990) presented a “Resilience Model,” in which a person faces adversities reacting to them for later reintegration of the lived experience, that is, the premise of the model is that, for a person to become more resilient, he/she must face challenges and risks that induce stress, having moments of disorganization and reorganization, learning from the experiences to further strengthen their coping skills and protective factors. This conceptual model shows how a person passes through adversity, starting from his bio-psycho-spiritual homeostasis (i.e., "comfort zone") and resorting to protective factors, such as the existence of a family support network, good self-esteem and great self-confidence (Richardson, 2002, 2011). Subsequently, the reintegration of this (un)conscious experience causes one of the four results: 1) dysfunctional reintegration, when the person does not overcome the adverse situation, reacting with risk behaviors (e.g., addictive behaviors such as alcohol and drugs or aggressive behaviors); 2) reintegration with loss, when the person has the desire and motivation to overcome adversity, but suffers losses (e.g., self-esteem); 3) reintegration and return to homeostasis, characterized by recovery of homeostatic balance, without any gain (i.e., has not really learned from the experience and will probably go through similar problems again until he learns from the life event); or 4) resilient reintegration, when there is growth and increased resilience, that is, the person benefits from positive growth as a result of lived learning (Richardson, 2002; Richardson, Neiger, Jensen, & Kumpfer, 1990). This fact leads to a better capacity of preventing or precluding the onset of pathological symptoms (Lester et al., 2010). In this perspective, we can also mention that resilience is seen as an inner strength which drives personal growth (Richardson, 2002, 2011), because in the case of military families, living similar experiences in the past may enable them to be more prepared for future situations. Therefore, despite experiencing stress inducing moments during a mission, it is important to note that military families have demonstrated resilience and growth after a mission. Thus, reformulating situations from a positive perspective can be a valuable coping mechanism (Ebata & Moos, 1991). As mentioned by U.S. Army (2007), one of the factors that seem to have the greatest influence on the reactions of the children to changes inherent to a mission is the coping strategies used, which may not be effective. The coping concept is defined by Lazarus and Folkman (1984) as a process, a "way to deal with" with adverse situations, where the cognitive-behavioral effort of a person subsists to mitigate the external and/or internal pressures that are evaluated by it as something beyond their resources. This process is active and may change with evaluations obtained on the events (Lazarus & Folkman, 1984), and with the developing age in which the person is found (Skinner & Zimmer-Gembeck, 2007). Coping represents a non-passive attitude of the person to demands to (re)balance, recover and prepare for new challenges (Skinner, 2007).

Skinner, Edge, Altman, and Sherwood (2003) identified 12 "coping families" of higher-order: problem solving, information search, helplessness, escape, self-confidence, seeking support, delegation, social isolation, accommodation, negotiation, submission and opposition. Each "family" includes more than one way of dealing with adverse events, considering these forms of lower-order. The "coping families" can complement each other because the answers are innumerable, adapted to specific demands and modeled by existing context and resources (Skinner, 2007; Skinner et al., 2003). Therefore, the results that contribute to resilience are resulting products from effective coping strategies, the existence of protective factors, as well as successful adaptations in past events, and the absence of internal disturbances (Richardson et al., 1990).

Younger children of school age are likely to use strategies that reflect internalization behaviors, reflecting in emotions like anxiety and fear, in an increased sensitivity to media coverage, and a reduced school performance. For Watanabe and Jensen (2000) adolescents may be more resourceful in coping strategies than younger children. Adolescents who take on domestic responsibilities become more independent, and their extracurricular and social activities with their peers are more reduced. They are also more likely to have a decrease in academic performance and an increase in depressive symptoms and behavioral problems due to the emotional stress inherent to the period they are living. A study of military children aged 11 to 17 revealed that adolescents, especially girls, reported more difficulty in school performance, family and peer relationships than younger children (Chandra et al., 2010).

Based on the Bioecological and Resiliency Theories, Wooten (2013) presented the "Deployment Risk and Resilience Model" (DRR model). Adapted from the resilience model of Richardson et al. (1990), the DRR model has a structure that helps to understand the risks of a mission, the protection factors, the homeostasis imbalance, the reintegration, and the final result in the end of the post-deployment phase. This model, when presented and adapted to the theme under study, focused on the service members and his specific context (organizational and social), suggests that both can influence the characteristics of the bio-psycho-social trajectory, and the reintegration after a mission cycle (Wooten, 2013). Being so, the unit of analysis is the service members, and the hypothetical relationships are transactional and interdependent, implying a process of mutual causality (Wooten, 2013). In the DRR model, resilience is conceptualized as a dynamic construction involving transactions between person-process-context-time (Richardson, 2002). Since the DRR model mirrors life events throughout a mission, that is, positive and negative experiences lived by the service members, from the deployment up to the reintegration of these experiences after the mission (Wooten, 2013), it can help to understand the intensity of the impact of a mission on the children of the assigned and deployed military. The importance of the reintegration process after post-deployment is shaped in the model, helping to understand the reintegration trajectories and their effects of equifinality and multipurpose (Wooten, 2013). Briefly, the DRR model provides for this study a conceptual bio-psycho-social framework of evaluation for future interventions with military families, especially with their children.

## The Portuguese Military

Portuguese service members have been participating in United Nations operations since 1996. For decades, they have been present in so-called "new missions" in TO such as Bosnia, Kosovo, Timor, Lebanon, Afghanistan and Iraq. In 2014, some studies began to be conducted with members of Portuguese military families, where the need for institutional support was mentioned (e.g., Barbudo, Francisco, & Santos, 2014), but few resources and support from the Portuguese military services aimed at Portuguese military families were developed only in the last decade, such as the Portuguese Army's "I, You & We" project (Barbudo et al., 2014). Despite this fact, the families of Portuguese service members continue to mention the need to implement specific programs for military families (Duarte, Francisco, Ribeiro, & Santos, 2020) because they identify gaps in military service support (e.g., Bóia et al., 2018; Duarte et al., 2020).

## Method

The general purpose of this exploratory and qualitative study is to understand the impact of an international military mission on the children of the deployed military. Specifically, it intends to answer the following questions: 1) what were the most critical moments for the children during a mission; 2) what perceptions of the children regarding the family and individual changes, felt during an international military mission of one of the caregivers; 3) what coping strategies were used by the children during this period (considering its “coping families”); 4) what were the outcomes in every phase of the mission; and 5) what outcomes identify the trajectory of the reintegration after the mission.

### Participants

Twenty-two children of the service members of the Portuguese Army participated in the study (13 boys and 9 girls) aged between 8 and 21 years old (*M* = 13.95, *SD* = 3.30), who, at the time of the mission, aged between 6 and 18 years old (*M* = 11.41, *SD* = 3.79). From the total participants, only two had no siblings at the time the military parent carried out the mission. All service members caregivers were male and have participated in one to six international peace support missions (*M* = 2.36, *SD* = 1.14), deployed for about 6 months in Afghanistan, Timor, Lebanon and/or Kosovo.

### Procedure

A purposive sample was used. The participants were selected based on the fact that, at least, one of the caregivers was a service member, who participated in an international mission. After the authorization of the Chief of Staff of the Portuguese Army, contacts were established with the various units of the Army (Mainland Portugal, Madeira and Azores Archipelagos), through one of the authors (the psychologist-lieutenant-colonel of the Portuguese Army). The service members who met the above criteria were subsequently contacted with the intention of asking their permission to interview children. The interviews, with an average duration of 55 minutes, were conducted at service members’ homes or military units. After consents were signed by the parents, and after verbal consent of their children, the interviews were recorded in audio. The interviews conducted with children aged between 6 and 9 years old were supported by drawings identifying moments related to the missions (e.g., time of the notification, moment of departure, thinking about the military caregiver during the deployment and the moment of arrival), in order to facilitate communication, being considered as a conversation tool (Pires, 2015).

### Measures

#### Semi-Structured Interview Guide

Taking into account the exploratory nature of the study, the interview guide, specifically designed for a doctoral project in which this study is inserted, consists of four thematic blocks, informed by previous research and the existing literature on this topic: A. Pre-Deployment, B. Deployment, C. Post-Deployment, and D. Experiences comparing missions and perceived learning. The blocks A, B and C sought to explore similar themes, such as, adaptation to change (e.g., “What else helped you deal with the changes during this period?”), expression of thoughts and feelings (e.g., “What difference did you feel in the way you expressed your affections to your parents? Did you get closer to him?”), family activities and leisure activities (e.g., “Have there been any changes in your daily life in your leisure time and family activities?”), change of responsibilities and family routines (e.g., “What changes have you felt in terms of responsibilities and routines?”). Block D, besides comparing missions lived by those who had more than one experience, it also sought to identify advices they would give to other children of the same age who could go through the same experience (e.g., “If you could give advice to a child who knows that his or her father/mother has been appointed to the mission, what would you say?”).

#### Socio-Demographic Questionnaire

It was built specifically for this study to gather socio-demographic data related to the mission of the service members (e.g., age, household and academic qualifications).

#### Data Analyses

After transcription, the interviews were submitted to the thematic analysis procedure, considered a flexible approach to analyzing qualitative data (Braun & Clarke, 2006), using QSR NVivo 10 software. The four main categories were previously defined, based on previous studies, the interview guide, and the specific research questions. After the identification of topics, the units of meaning were organized into specific categories whenever a given pattern was noticed (Braun & Clarke, 2006). The categorization was performed through a deductive-inductive process in which the thematic categories that emerged in the text where also compared with the existing literature and the four main previously defined categories (Daly, 2007). The credibility and validity of the research was also assured by triangulation of researchers (Miles & Huberman, 1994). Therefore, raw data responses were individually identified and grouped into themes and subthemes developed by the first author and checked for reliability and validity by the other authors until a consensus was reached.

## Results and Discussion

The thematic analysis resulted in an interrelated hierarchical system with four main categories: mission phases (pre-deployment, deployment and post-deployment); family and individual experienced changes (examples of subcategories: routines, responsibilities), coping strategies used (examples of subcategories: accommodation, information search, troubleshooting) and trajectories of reintegration perceived during the phases of the mission (examples of subcategories: impact of the mission, advice to be given to other children). The following subsections report our findings on the referred categories, presenting the number of participants who referred to each theme. The citations presented, illustrating the content codified in the respective categories, are accompanied by the identification of the participants, by sex (male, M; female, F), number and age, thus ensuring the confidentiality of the data (e.g., M3, 17y).

### Mission Phases: Most critical moments

This category is associated with all other categories and subcategories, because it allows to distinguish the specific aspects of each theme under analysis, according to the moment of the mission cycle which the participants referred to. However, the "most critical moments of the mission" emerged as a specific subcategory and in this regard the pre-deployment (17 participants [p]) and the deployment (16p) were considered as critical moments, which is in line with results of previous studies. For example, the National Military Family Association (NMFA, 2005) showed that the most stressful moments of military families were the notification, the moment of departure of the service member, and the actual deployment. In our study, regarding the pre-deployment, the moment of the notification stands out and sadness was the emotion that most prevailed (10p), accompanied or sometimes replaced by surprise (5p) or by irritability (3p). These emotions were prompted mainly by the fear that something might happen to the military caregiver (7p). This fear increases if the TO is "of war" and if children are more aware of the dangers that can arise from it (Andres, 2010).

I was not expecting it the first time. Of course I was upset because I did not want it… I was 11 or 12 years old (M5, 15y).

It was more difficult because it was in Afghanistan, but when I was younger it was even more difficult, I did not understand very well. Yes, this time I was more afraid (F9, 18y).

It is interesting to notice that nine children really appreciate the way the notification is made, being important to explain and clarify the situation so that it makes sense, what is in agreement with literature (e.g., U.S. Army, 2007). Participants also consider that the notification should be made as early as possible to avoid biased fantasies (mainly caused by the news about the wars that appear in the media), and with the presence of both caregivers so that there is a feeling that they are all together in this decision.

They must have talked to each other about how they should tell me the news, and afterwards they talked to my brother and me, (…) my father explained the reasons why he was leaving, my mother helped him to convey the message, and this prevented us from entering into a state of shock (M5, 15y).

This particularity is also referred to by other authors (Bóia et al., 2018), who consider important the service member to explain, together with the spouse, what will happen, the reasons why the father is leaving, what he will do, etc. This is because, after the notification, there are doubts and concerns associated with expectations about the prolonged absence of the military caregiver. However, the perception that something is going to change sometimes precedes the notification itself (5p).

He himself gave me the news, I had also perceived it by his conversations with my mother (M5, 15y).

Still during the pre-deployment, the last days and the farewell were also considered as very difficult moments (8p), what is in agreement with literature (NMFA, 2005).

It was more difficult, to see him leaving … And me staying here, because I was thinking a lot about Dad (F3, 8y).

However, it is the beginning of the deployment that is identified by most children (15p) as one of the most difficult periods due to the need to adapt to the physical absence of the service members, new routines, and increased responsibility. At this stage, feelings of nostalgia, sadness and anxiety are added, and can last up to 2 months (6p), school performance was affected at this stage (3p), and there was an increase in conflicts with the at-home caregiver (2p teenagers).

Yes, there were two months when I had to do everything. Then they would say to me "do this, don’t do this" and I would immediately react, there was nothing else to do, for some reason, I was also very nervous to have a lot of responsibility on me, and it was difficult for me (M10, 21y).

These reactions caused by the physical "loss" of the military caregiver are found in several studies which report that changes felt together with intense negative emotions provoke behavioral outcomes, such as, anger projection and verbal aggression on others (Huebner, Mancini, Wilcox, Grass, & Grass, 2007), as well as changes in school performance (Misra & Singh, 2014). However, the last days of the deployment were also very emotional for 15 children, because anxiety, nostalgia, and joy are triggered by expectations raised by the post-return.

(…) we were always stressed out. "Is he coming tomorrow?" and that was when "ah!, he is coming in two or three days," "is he already on the plane?," no! "do you already know when you will get on the plane?," me too. On the following day more two or three days were added, we were even more nervous (M10, 21y).

The results of this research are in line with some studies (e.g., Knobloch et al., 2015; NMFA, 2005), partially differentiating them from others, such as Chandra et al. (2010) which revealed that children, in addition to having had more difficulties in deployment, also felt it in the post-unemployment, due to the addition of new responsibilities. Also Sheppard, Malatras, and Israel (2010) consider pre- and post-deployment as the most critical phases. These differences in results between the various studies may be due to multiple variables, such as, the nature of the mission, the danger in the TO, the individual characteristics of the children, and the idiosyncrasies of the systems where they are inserted. Also, regarding their age, children report greater difficulty of adaptation at earlier ages, because they have little awareness of reality, so causing confusion, surprise and sadness projected in irritability behaviors, seeking attention and affection (Van Breda, 2001). Grown up children (i.e., adolescents) are more aware of the potential risks involved, the concerns, and the real well-being experienced in the moments lived (Andres, 2010).

Another aspect that we believe is relevant to highlight is that during the deployment there is a possibility of the service members having leave days, which is seen by the children as a good thing, although later on it will be difficult to say farewell "again."

Yes, it is very important for us, but on the other hand it is so difficult, because it will be another separation… but the good part stays (F9, 18y).

### The Changes Experienced During the Mission

#### Pre-Deployment

Upon notification, simultaneously with the raising of expectations about "how it will go," participants reported they were given advice, mainly from the service members, and a small "training" according to the responsibilities of their new domestic tasks. These are intended to prepare the family for the deployment phase, which is in agreement with literature (e.g., Pincus et al., 2001).

(…) When my father gave me the news he said, "Let's start doing it. I will help you too, but you are going to have to get used to it so it will not be so hard when I am gone.” And that was important. It was very important (M5, 15y).

"Now you are the man of the house," "you are the eldest" or " take care of your mother now" are the expressions given to older children of the family, sometimes producing feelings of ambiguity in roles and responsibilities (Huebner et al., 2007), or even a sense of inability to fulfill the imposed "mission." These ideas were also identified in other studies (e.g., Misra & Singh, 2014; Paley et al., 2013), although it is considered a practice to be avoided (Pincus et al., 2001; Van Breda, 2001).

Be the man of the house? I thought I was not going to measure up. I even thought "hey! now I have to deal with things, now I have to take care of something, my mother is not here, Filipe is leaving, Filipe is leaving” (M10, 21y).

#### Deployment

During the deployment, 17 children perceived and felt changes mainly related to the increase of household tasks (12p), such as, helping in the kitchen (10p), tidying up and cleaning the house (8 p), taking care of pets (4p) and cleaning outside the house, the pool and the garden (4 p). However, they have also been reported changes in study support (11p) and between siblings (10p), since the young ones have acknowledged the help of their older sibling(s), especially the firstborn who assumed himself as the substitute of the service members. All these results are consistent with other studies (e.g., Chandra et al., 2010; Huebner et al., 2007; Knobloch et al., 2015; Misra & Singh, 2014).

Maybe my father made my brother my second father, so to speak. My brother (…) said "you have to study," to this day he says "do not forget to study," and I think that this adjustment of position … helped me to get through this situation (M5, 15y).

According to existing studies (e.g., Barbudo et al., 2014; Knobloch et al., 2015; Misra & Singh, 2014), it was reported by 10 children that there was an increase of "responsibilities and duties" for the at-home caregiver. The change in parental responsibility, along with the increased burden of duties for the at-home caregiver, also implied a greater sense of freedom, essentially felt by the older children (4p). This fact is also referred to in the study by Misra and Singh (2014) in which young adolescents report that the absence of the military caregiver meant greater relaxation, greater freedom and fewer restrictions. However, this feeling of greater freedom goes against the study of Huebner et al. (2007), in which adolescents describe a greater "prison" caused by new routines and responsibilities.

Then when he went on mission, we had too much freedom, we had a lot of time alone because my mother could not always be there… (M1, 18y).

At first it affected a lot because my father is more rigid than my mother regarding obligations. My mother says 'go to study', and sometimes I would go and sometimes I wouldn’t… it is not like this with my father (M5, 15y).

#### Post-Deployment

During the post-deployment, the majority of the participants (12p) identified the need for readjustments, both from the service member and the family, so that the reintegration of the service member into everyday family life was as natural as possible.

(…) one can notice a little bit his natural effort. It lasted six months and we had a very fixed routine, so I guess it costed him a little bit because he came from there, but it was quick [the readjustment] (F9, 18y).

This readjustment after the return is referred to in existing literature, which evidences the existence of challenges of reintegration and renegotiation of roles and/or rules, both for the service member and the family (e.g., Andres & Coulthard, 2015; Huebner et al., 2007). This means that, despite the stressful challenges inherent in this phase, the availability of the resources used to communicate during the displacement with the service member (the video call stands out) contributed to maintain the connection and permanent closeness, continuing a secure bond, which subsequently enhanced the ease of reintegration after their return and is also described in the literature (DeVoe & Ross, 2012).

### Coping Strategies

#### Pre-Deployment

The results showed that accommodation (e.g., focus on the positive, acceptance) was one of the coping strategies most used by the children to deal with the mission of the military father in the pre-deployment phase (9p), especially in the period prior, during and after the notification. This can result in positive thinking about the situation, in cognitive restructuring, and in minimizing the problem, or simply in acceptance (Skinner & Zimmer-Gembeck, 2007). This flexibility can be the product of past experiences which leads to see the world as predictable and consistent (Van Breda, 2001). In fact, eight participants reported that the previous experiences of separation for the same reason are a source of emotional stability, a built-in feature that acts as a protective factor (e.g., Riggs & Cusimano, 2014).

It was easier because I was used to another mission, this went faster and I already knew what I had to do (F16, 13y).

I think I can help because I've been through this experience, as well as my friends who had already had this experience and who helped me. (F21, 15y).

Several studies converge with these results, and even a few reported that the children of the deployed service members manifest a high level of resilience (e.g., U.S. Department of Defense, 2010). Another strategy widely used after the notification was the search for information (20p), a strategy that is widely reported in literature about adolescent’s coping skills in general (Skinner & Zimmer-Gembeck, 2007). Although they admit that they have avoided talking about the mission (7p), knowing the TO where the service member will be deployed, and what he will be doing are examples of information that the children want to have. This happens through the Media, and the reading of other existing resources, which demonstrates the importance of the existence and accessibility of appropriate information to the ages of the children.

After we had finished our meals, and instead of everyone going to their room and being on the Internet, we would sit on the couch and talk … and we wouldn't talk about him leaving, because it was more difficult for him (M1, 18y).

[Information from the Portuguese Army's "I, You & We" project] … can always be a help for families to prepare and know. I think it's funny, the vision of Kabul, climate, temperature… I think it helps in the interaction of families (F1, M17).

The search for support during the pre-deployment was also a strategy widely used by the military children (13p). This strategy, according to Skinner and Zimmer-Gembeck (2007), is one of the most used in all ages. Being influenced by the development and by the chronological maturity, this form of strategy may be different either by the source of support required (e.g., parents, peers, teachers), either by the domain (e.g., doctor, academic), by the type of support sought (e.g., contact, comfort, guidance, instrumental assistance), and/or by the way the support is sought (e.g., distress, social references, proximity, verbal requests). In this study, the children reported that the main sources of pre-deployment support were parents and peers, and the type of support sought was essentially comfort through greater proximity.

Friends were also very important during the pre-deployment (…). My family got closer during the pre-deployment… (M5, 15y).

Before he left, I spent more time with him, to enjoy him, I was already feeling that I would be missing him … not so much, but giving him love all the time, and at that time even more (F20, 12y).

Generally, the use of the problem-solving strategy also differs according to the age of the children. While younger children sought to solve problems through a combination of strategies, which may include seeking adult support, older children used mostly instrumental actions and building action plans (Skinner & Zimmer-Gembeck, 2007). Also during pre-deployment, the perception and feeling that routines and responsibilities should be kept is referred as relevant by 13 children, being essential for individual and family balance, what is in agreement with existing literature (e.g., Pincus et al., 2001; Van Breda, 2001), thus being a very important coping strategy for the children of the deployed service members.

I tried not to change too much to be able to distract myself too. The routine was very important (M5, 15y).

Another way for military children to face the challenges of the mission it is the avoidance (which may be behavioral and/or cognitive avoidance, denial attempt or illusory thinking; Skinner & Zimmer-Gembeck, 2007), as reported by seven children. This strategy is sometimes supported by carrying out activities to occupy "the mind and the heart" (e.g., computer games, listening to music), or seeking support from the family or social network, and it is sometimes opposed to a control strategy which reflects a more conscious approach to adversities to adopt preventive measures later (Latack & Havlovic, 1992).

I listened to music, played games, went out with my friends, hung out with them, I avoided talking about these things. In part it worked, but we were always thinking about it. There is a time of the day when we would think more (M1, 18y).

The self-confidence, materialized by an emotional and behavioral "control" to allow a greater restraint in the emotional expression, was mentioned by six children as a coping strategy used in the pre-deployment.

To deal with the situation I … I do not get worried before things happen, I get worried when they happen. It will take time (F20, 12y).

Finally, other strategies were used during the pre-deployment phase, although being mentioned by less participants: feeling of helplessness (confusion, interference and/or cognitive exhaustion), as perceiving the limit of capacity (3p); opposition strategy, through guilt projection and aggressive behaviors, fundamentally verbal (2p); and negotiation strategy through persuasion (1p).

I was not expecting it the first time. Of course I was upset because I did not want it… I was 11 or 12 years old (M5, 15y).

If there was news “Attack kills I do not know how many in Afghanistan”… that's it! "hey!," It would start right there, the theme was “maybe you should not go…do not go, do not go.” It started like this (M10, 21y).

#### Deployment

Regarding the deployment, two coping strategies were mentioned by all the children: the search for support, which reinforces the idea of being one of the strategies most commonly used by all ages; and problem solving, which reflects the concern of carrying out effective actions to mitigate the stressful situations, whether they are instrumental, strategic or planned (Skinner & Zimmer-Gembeck, 2007). In relation to the search for support, relatives (especially the nuclear family, including the physically absent service member), colleagues and teachers, and friends were the sources of support to encourage and help during this stage of the mission, according to existing findings (e.g., Chawla & Solinas-Saunders, 2011). Riggs and Riggs (2011) refer that the literature suggests that a secure bonding system between the at-home spouse, the service member and the children, is a protective factor that promotes strategies for effective coping during deployment and post-deployment.

Essentially I shared my problems with my mother, also with my friends because I do not like to keep so many things for me… (M1, 18y).

My mother spoke with the head of the class who spoke with the teachers. I think the school has an important role during the period of deployment (F5, 15y).

To talk to him on the computer and to know he's okay (M14, 15y).

At the deployment stage, communication between the children and the service member resulted in an emotional support, a collection of advice and/or a sharing about everyday life (e.g., by the children about school and extracurricular activities, by the service member about work and daily life in the TO). This communication was mostly interactive or direct, especially through video calls (19p) and the telephone/cell phone (6p). Late communication (e.g., letters, packages and emails) was identified by four children as a resource of high satisfaction, as it is embodied in something concrete and tangible, providing repeated support, and the possibility of "turning to" several times (e.g., Barbudo et al., 2014). This almost permanent contact allows the service member to be present psychologically, even if he is physically absent (Andres & Coulthard, 2015).

During the deployment I did not have difficult phases. It was very easy. I do not know, we talked every day… Skype is fantastic, we can see each other! (F21, 15y).

I wrote two letters and received one. It was good to feel a little bit relieved (F21, 15y).

Routine is by itself a fundamental strategy to "normalize" and balance the daily life of the children during the period of the service member absence (Riggs & Cusimano, 2014). If keeping the routine during the pre-deployment was a concern for the children, during the deployment this issue is of utmost importance. This problem-solving strategy is essential during the absence of the service member. Maintaining or restoring routine has the positive consequence of making daily life undisturbed, or, if these exist, to be of minimal impact (e.g., Knobloch et al., 2015; Paley et al., 2013).

If we change the routine drastically there may be major consequences for the person (M10, 21y).

(…) there was communication during the deployment. As if it was normal. We have not done anything new. We have kept our routine, but without him! (F21, 15y).

The cognitive-behavioral effort of the children also involves implementing other strategies, such as, accommodation (10p), that is, distraction, cognitive restructuring, minimizing lived circumstances, or simply acceptance; but also the escape (12p), mainly materialized in mental withdrawal.

I was always worried about my father that something was going to happen, so I tried not to think about it, I tried to think about other things (M5, 15y).

I began to get used to it … First I felt sad and then I started to feel happy, because I knew my father was well and I could talk to him every day (M17, 10y).

The search for information was mentioned by most of the participants (17p), mainly through the service member himself. Therefore, direct communication with the service member is essential, not only to find support, as previously mentioned, but also to know about his situation, and news are essential to calm down.

He said he was helping that country to achieve peace. (…) I thought he was making peace. I felt happy (F3, 8y).

Another strategy recognized by six participants, especially the youngest, was the perception of helplessness. As Skinner and Zimmer-Gembeck (2007) refer, this type of strategy may be used by children with less solid bonding to their caregivers and/or experiencing the separation anxiety for the first time, in this case because the military caregiver is deployed. The feeling of insecurity due to the absence of the primary caregiver who provides security and shelter, it is also described in the study of Misra and Singh (2014).

When I was younger, I thought that I would not see him again … (F4, 11y).

I missed him more, because he was not here, and then I began to feel his absence. At night I move around a lot and hit the wall for my dad to say, "Come on, make less noise." [Did you keep hitting the wall even with your father absent?] Yes, to keep myself calm (…) because I like to fan myself… and I hoped my father would come (M17, 10y).

Finally, some of the military children (3p) mentioned that they had some opposition behaviors, as a challenge or a manifestation of anger towards the caregiver who left them. This behavior is also referred to in other studies with children of the deployed military (e.g., Knobloch et al., 2015).

(…) when I was very, very sad, I would do things [parental opposition behaviors because I was angry that the service member was absent], and Mom would be angry at me, but then it would pass (F3, 8y).

(…) other people say “Oh, poor mother, your father is not here, you have to help your mother, now that your father is not here"… I did not like it, but I wouldn’t think "Oh, poor me"…no! He simply was not here, that's it! [manifestation of anger about her father not being with her] (F11, 17y).

The emotions most associated with post-deployment phase, especially the moment before the arrival, were anxiety (9p) and joy (21p) with the return of the service member, meeting the existing literature (e.g., Pincus et al., 2001; Riggs & Riggs, 2011).

I was anxious and happy, I knew it wouldn't be long, he was going to stay with me (F4, 11y).

#### Post-Deployment

For most of the children, the readjustment after the return of the service member lasted no longer than 1 month. This perception may indicate that there were factors that contributed to minimize the impact of the mission on children, such as activities carried out together (e.g., leisure activities, talking, eating out).

Despite being a bit difficult to go back to what it was before, I think it was easier for me to adapt, than to adapt myself to his absence. But a month later everything went naturally (M1, 18y).

These results are in agreement with some studies that show that, although the children exhibit some behavioral changes during the deployment of the service member, most seem to adapt quickly to the reunion (Jensen, Martin, & Watanabe, 1996). Possibly, for this quick and easy readjustment of the family system, "easy and permanent" contact with the service member may have contributed throughout the deployment (Riggs & Riggs, 2011).

### Outcomes and Reintegration

In pre-deployment, 16 children referred that they got close to the service member and the nuclear family, sometimes aiming to alleviate the suffering and fears of their parents. During the deployment, 20 children mentioned a closer relationship to the at-home caregiver, and the siblings, while being careful to stay "connected" to the service member. Already in the post-deployment, 16 children also mentioned that they got closer to the service member after his return, restoring this relationship (Riggs & Cusimano, 2014; Riggs & Riggs, 2011) and even reinforcing it in some cases.

Through conversations, hugs and cuddles we were also reassuring him before going on mission. So… not to be very difficult for him (M1, 18y).

[Deployment] made the bond between my brother, my mother and me much stronger (…). My father has been adapting, and the relationship… of course it has grown! And now it is much better (M5, 15y).

The perception of closeness to the elements of the nuclear family, throughout the mission is in agreement with previous studies (e.g., Knobloch et al., 2015). During the interviews, when asked if they would be able to help/advise other children/youth who might be going through the same situation, 21 children said yes, sharing possible advice. During pre-deployment the children advised the other children/youth not to be sad, to be strong and not to worry (12p), to spend more time with the service member and the family (6p), and to do activities that they like (5p). During the deployment, they advised them to talk to the service member, preferably by videoconference (11p), not to worry so much, and to distract themselves (9p), to seek support in friends, teachers and family (8p), and to maintain the routine (3p). Finally, in the post-deployment, they advised, as a priority, to be close to the service member to catch up.

Before his father leaves, he should spend a lot of time with him and to make the most of it (F16, 13y).

To spend time playing, forgetting every day, and one day Daddy comes back (F3, 8y).

During the post-deployment? Enjoy! (F21, 15y)

Being able to advise peers can be a sign of personal growth or adaptation (resilient reintegration), and not just the recovery of their condition before the mission. As mentioned before, resilient reintegration is a process in the presence of adversity that results in growth (Richardson, 2002), being referred to by Wooten (2013) regarding the deployed military. In the same sense, in addition to the negative effects identified in our findings and in existing literature in this study we also point out that the children perceived positive changes in their life after the mission(s), attributing new meanings to the experience lived. In addition to re-connecting to their family, they felt an increase in their own responsibility (4p) and in the attribution of tasks (8p), some of them embodied in new routines, and they also recognized their maturity gain (10p). These testimonies are in line with some literature (e.g., Lester et al., 2010; Park, 2011; Riggs & Riggs, 2011) que suggest that most children show evidence of resilience over the course of the deployment cycle.

I agree that the missions help us to be prepared (…) It did no harm (M12, 18y).

I think that if there was no mission I would have also grown up, but with the absence of an important person, it makes us grow up faster, and have responsibilities that I didn’t have (F21, 15y).

## Conclusions

The present study showed that the military children trigger a set of strategies and resources (protective factors), with the purpose of minimizing the adverse events inherent to each one of the phases of the mission, which can contribute to a resilient reintegration, as referred by several authors, depending on individual characteristics and skills and experiences lived (Rutter, 1999). During the presentation of our findings, we highlight the variability of coping strategies by children throughout the phases of a mission, which is an interesting finding of our study. The most critical moments of a mission were mainly identified as the notification period and the last days before the departure of the service member, as well as the phase of deployment, especially the first weeks. This is caused by changes in the routine, the increased responsibility, the freedom, and the domestic tasks, due to the absence of an important element of the nuclear family, which is in agreement with literature (e.g., Knobloch et al., 2015; Misra & Singh, 2014; Pincus et al., 2001). Regarding the changes perceived during the pre-deployment, the children felt they were given advice and "training" for the next phase. During the deployment the perceived changes are related to the increase of tasks, the closeness between elements of the family, and the younger ones recognizing the support of their older siblings, feeling that these are the substitute of the absent caregiver. During the post-deployment there was the perception that there was a concern in the readjustment of the family system, so that it was as natural as possible. In the beginning of the deployment, as well as in the post-deployment, the children seem to be adapting quickly to the situation lived (Jensen et al., 1996). The coping strategies most used by the children were the search for support from their peers, family members and teachers (e.g., Chawla & Solinas-Saunders, 2011; Knobloch et al., 2015; Misra & Singh, 2014), and problem solving. However, and especially during the pre-deployment, the search for information was also a practiced strategy. Finally, and as outcomes, it was mentioned that the reintegration of the lived experience allowed a greater closeness between the family, especially the nuclear one, and an increase in responsibility, maturity and personal growth. Not only looking for the risk factors, we attempted to highlight some strategies used by the service members' children in the most critical moments of a mission, through first-person narratives. As this kind of studies are scarce, our findings, pioneers with children of Portuguese military personnel, offer a set of guidelines that may help military families, especially children, to structure their mission experiences in terms of strengths and gains, instead of focusing only on negative effects (Knobloch et al., 2015). As Park (2011) points out, if military families, in general, have done well in the past, then in the present, they should seek to disseminate it as a source of pride and inspiration.

The study presents some limitations that must be taken into consideration when reading the results, such as, the range of the age of the participants given the sample size. On the other hand, the results do not reflect the perspectives of several family subsystems (e.g., parental subsystem), and they only report experiences of the children of the male service member of the Portuguese Army, which should be rectified in the future studies, namely through comparative studies of various branches of the Armed Forces. These studies should be extended to the whole family system, in order to better understand the strategies and resources that can contribute to the readjustment of children (and parents) during a mission. Some practical implications should also be considered. Taking into consideration that the children of the deployed military prefer the informal support (e.g., family and friends), it will be important to create informal social support networks which address the specific needs of the military families, especially in the deployment phase (Andres & Coulthard, 2015). However, other entities (e.g., religious, military, school) will also be important in supporting the children during all phases of the mission (Flake et al., 2009). The branches of the Armed Forces should explore and materialize the results of studies carried out with the Portuguese military families using existing forms of support (e.g., Intervention and Psychological Support Model provided by the Center for Applied Psychology of the Army), also enriching them with positive adaptation to international military missions. By analyzing the positive coping strategies of the service members' children, we can expand the existing resources of support to military families, emphasizing that the focus of what has resulted is not underestimated or ignored what has gone wrong (Park, 2011). It would also be important to promote social cohesion between organized military units and families, through family support centers, whose core work consists of immediate provision of services (e.g., spiritual, bureaucratic and peer support), or referral to other services. Such interventions will help military families to successfully adapt to the demands of the military life, especially during missions, at which time the challenges can be more severe (Andres & Coulthard, 2015), so enhancing protective factors and, at the same time, reducing risk factors.

## Data Availability

The Semi-Structured Interview Guide and the Socio-Demographic Questionnaire developed for the study can be requested directly from the authors.
